# Functional Magnetic Resonance Imaging Connectivity Analyses Reveal Efference-Copy to Primary Somatosensory Area, BA2

**DOI:** 10.1371/journal.pone.0084367

**Published:** 2014-01-08

**Authors:** Fang Cui, Dan Arnstein, Rajat Mani Thomas, Natasha M. Maurits, Christian Keysers, Valeria Gazzola

**Affiliations:** 1 Department of Neuroscience, University of Groningen and University Medical Center Groningen, Groningen, The Netherlands; 2 Netherlands Institute for Neuroscience, an institute of the Royal Netherlands Academy for Arts and Sciences, Amsterdam, The Netherlands; 3 Department of Neurology, University Medical Center Groningen, University of Groningen, Groningen, The Netherlands; Brainnetome Center, & National Laboratory of Pattern Recognition, China

## Abstract

Some theories of motor control suggest efference-copies of motor commands reach somatosensory cortices. Here we used functional magnetic resonance imaging to test these models. We varied the amount of efference-copy signal by making participants squeeze a soft material either actively or passively. We found electromyographical recordings, an efference-copy proxy, to predict activity in primary somatosensory regions, in particular Brodmann Area (BA) 2. Partial correlation analyses confirmed that brain activity in cortical structures associated with motor control (premotor and supplementary motor cortices, the parietal area PF and the cerebellum) predicts brain activity in BA2 without being entirely mediated by activity in early somatosensory (BA3b) cortex. Our study therefore provides valuable empirical evidence for efference-copy models of motor control, and shows that signals in BA2 can indeed reflect an input from motor cortices and suggests that we should interpret activations in BA2 as evidence for somatosensory-motor rather than somatosensory coding alone.

## Introduction

The blood oxygen level dependent (BOLD) signal in premotor (PM) and, as recently described, primary somatosensory cortices (SI, Brodmann Area (BA) 2 in particular), is increased while participants perform actions and while they witness similar actions performed by others [1]–[3] suggesting a duality: witnessing others' actions triggers vicarious *motor* representations in PM and vicarious *somatosensory* representations in BA2 [4], [5]. This duality is prompted by reverse inference [6]: because electro-stimulation of PM can lead to overt movements and that of BA2 to somatosensory percepts [7] activations in the former are thought to reflect motor, and in the latter somatosensory processes.

Contemporary theories of motor control however suggest intensive crosstalk between motor and somatosensory regions [8]–[12]: each motor command sent to the body also reaches somatosensory cortices, as an efference-copy that forward internal models convert into expected sensory consequences [8]–[12]. The supplementary motor area (SMA) is considered the most likely source of the efference-copy [13]. The notion of efference-copy blurs the duality in the distinction between motor and somatosensory information and begs the question whether activations measured in BA2 in a variety of paradigms necessarily always represent somatosensory information alone or, at least sometimes, also (efference copies of) motor commands. Only very few studies have investigated this question.

Christensen and colleagues (2007) blocked sensory afference from the leg and compared the difference between active and passive ankle movements while the participant was or was not under the influence of ischemia. As expected, ischemia reduced SI activation during passive ankle movements, but this was not the case during active movements, suggesting that an efference-copy of the motor signal can determine activation of SI if actual somatosensory afference from the leg is missing or reduced [14].

Whether an efference-copy can significantly influence BA2 activation in the presence of normal physiological afference to BA2 however remains controversial. Two studies found no SI difference between the active and passive execution of a movement [15], [16] while one found smaller activation in SI during active compared to passive finger tapping [17].

**Figure 1:**
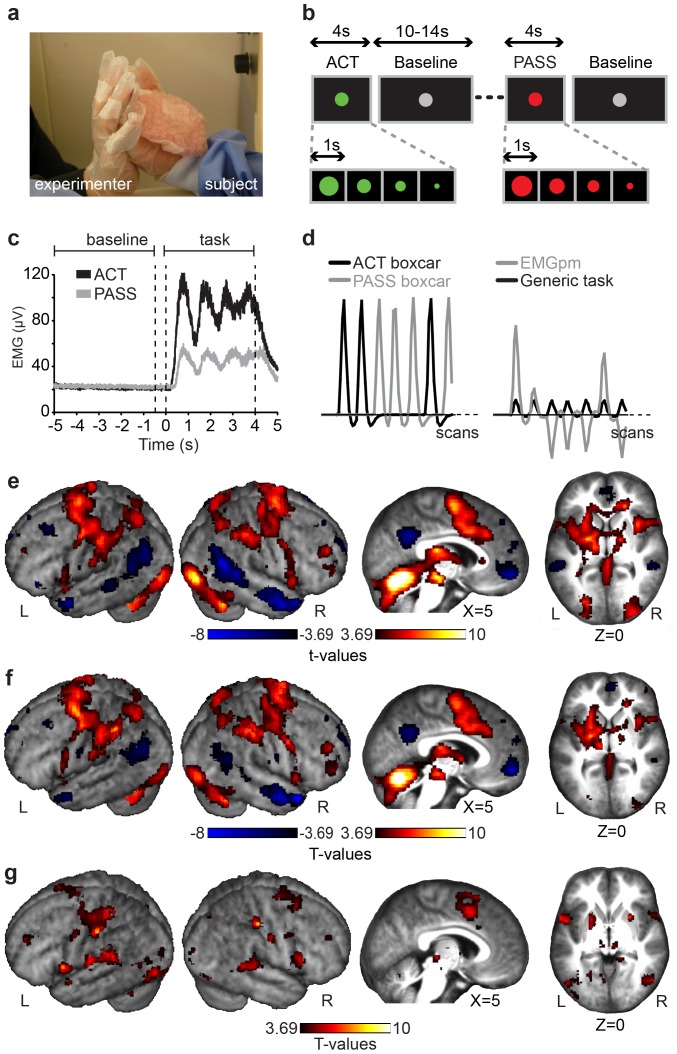
Experimental design and main results. (**a**) A photo of the experimental set-up. (**b**) Schematic diagram of the experimental design. (**c**) Grand-average EMG responses during ACT and PASS conditions. Time 0 marks the onset of the 4 s blocks. (**d**) Comparison of the standard boxcar approach (left graph) to the data-driven EMG approach (right graph) to modeling the fMRI data of a representative subject. In the standard approach, a boxcar predictor models ACT blocks and another PASS blocks. In the EMG approach, a boxcar predictor models the effects of a nonspecific, generic task (i.e. a single predictor models both the ACT and PASS blocks); and the standardized and mean-corrected EMG is included as a first-order parametric modulator (EMGpm) of the generic task predictor. (**e**) fMRI results of the comparison between the ACT and PASS conditions (**f**) fMRI results of EMGpm versus baseline. (**g**) PPI results (for e-g, voxelwise threshold: p_unc_<0.001; only clusters of at least 10 voxels are shown. All results also survive q_fdr_<0.05).

To provide further insights into this question, we compared participants' brain activity, measured with functional magnetic resonance image (fMRI) with their muscle activity, measured with electromyograhy (EMG) during active (ACT) and passive (PASS) squeezing (Fig. 1a and b). While during ACT trials participants gently squeezed bubble-wrap attached to the palm of the right hand, during PASS trials the experimenter pressed the subject's fingers around the bubble-wrap (see Methods for more details). Muscle activity was measured to quantify the intensity of the actual motor output and as a proxy of the motor command [18] and hence efference-copy signal intensity. By comparing muscle activity with brain activation, we investigated if SI activation can reflect the magnitude of the motor efference. In addition, we used two connectivity analyses to localize the likely source of this efference-copy and distinguish it from somatosensory re-afferences.

## Results

### EMG

**Figure 1c** **presents the average rectified (i.e. absolute value) electromyography (EMG) responses across subjects over a 10 s interval centered on the onset of the instruction to squeeze. The clear peaks and valleys of the EMG indicate good within- and between-subject consistency in the timing of the four squeezes.**

**For each trial, the rectified EMG during baseline (i.e. −5 s to −0.5 s relative to the onset of the task instruction) and experimental epochs (i.e. the 4 s of ACT or PASS) were averaged separately and the former average subtracted from the latter to yield baseline-corrected estimates of the EMG activity for each experimental trial. The baseline-corrected estimates for ACT and PASS where then averaged across trials to yield a single value per subject and condition, that were then compared using t-tests across participants. These values were greater than zero for both ACT (Mean = 77.18 µV; t_(17)_ = 6.88, p<10^−7^) and PASS (Mean = 32.04 µV; t_(17)_ = 3.25, p<0.002), and the difference between ACT and PASS was highly significant (t_(17)_ = 5.5, p<10^−4^). Accordingly, comparisons of brain activity in ACT and PASS trials are not of cases in which there was motor activity vs. cases in which there was none, but of cases in which there was more vs. less motor activity, and hence efference-copy.**

### General Linear Models (GLMs)

Two GLMs were then calculated for the fMRI data (Fig. 1d). In the first model a standard boxcar predictor was produced separately for ACT and PASS and convolved with the canonical hemodynamic response function (HRF). In the second model a single ‘generic task’ boxcar predictor was produced which contained both ACT and PASS blocks. In addition, a first-order parametric modulator was defined using the EMG (EMGpm). The value for a particular block was calculated as the average EMG during the 4 s block minus the average EMG during the preceding baseline (from −5 to −0.5 s of the appearance of the task instruction). The parametric modulator (EMGpm) was then demeaned and standardized, and both predictors (the generic task predictor, and the generic task predictor * the EMG) were convolved with the HRF.

Figure 1e–f show the fMRI results of comparing ACT versus PASS conditions and EMGpm versus zero. Both ACT>PASS and EMGpm>0 revealed widespread differential activations in areas typically associated with motor programming and execution including the cerebellum, primary motor cortex (M1), SMA, PM and the posterior parietal lobe, including area PF and the superior parietal lobule (Table 1). Most relevant for the present report, SI was activated in both of these contrasts, in particular its BA2 sub-region. In the EMGpm>0 analysis, both the left and the right BA2 showed significant modulation, with a larger proportion of the left BA2 (51.3% of the anatomical region of left BA2 was activated; contra-lateral to the squeezing hand) being modulated than the right BA2 (44.3% of the anatomical region of right BA2 was activated; ipsilateral to the squeezing hand).

**Table 1:** Clusters of activity resulting from the contrasts ACT>PASS, EMGpm>0, and the PPI analysis.

Cluster size in number of voxels	Number of voxels in CytoArea	Hem	Cyto/anatomical area	% of CytoArea activated	x	y	z	T
**ACT>PASS**								
33901	2132	L	Area 6	48	N/A			N/A
	1978.1	R	Area 6	44.6	N/A			N/A
	1463.3	R	Cerebellar Lobule VI (Hem)	76.4	6	−68	−18	17.77
	1396	L	Cerebellar Lobule VI (Hem)	69.3	−32	−56	−30	11.61
	725	R	Cerebellar Lobule VIIa Crus I (Hem)	21.5	N/A			N/A
	608.4	L	Cerebellar Lobule VIIa Crus I (Hem)	19.2	N/A			N/A
	580.1	R	Cerebellar Lobule V	70.1	2	−56	−10	14.31
	458.9	L	Cerebellar Lobule V	60.3	N/A			N/A
	446.8	L	Area 2	47.9	N/A			N/A
	409.5	R	Area 2	41.6	N/A			N/A
	375.7	R	Area 44	41.3	N/A			N/A
	368.7	R	hOC3v (V3v)	52.9	30	−84	−8	10.09
	339.2	L	Cerebellar Lobules I–IV (Hem)	67.5	N/A			N/A
	N/A	R	Middle Cingulate Cortex	N/A	10	10	34	10.1
	N/A	L	hlPI, BA3a, BA4a, BA4p	N/A	N/A			N/A
	N/A	R	hlP2, BA44, SPL(7A and 7P)	N/A	N/A			N/A
	N/A	L/R	hlP3, BA3b, Insula, Putamen, Pallidum, Thalamus	N/A	N/A			N/A
440	316.4	L	SPL (7A)	19.1	−16	−68	54	6
	52.3	L	SPL (7P)	9.6	−14	−68	58	5.65
	32	L	SPL (5L)	6	−14	−52	64	4.59
	N/A	L	Superior Parietal Lobule	N/A	−18	−66	58	5.74
	N/A	L	Precuneus	N/A	−16	−58	66	4.13
90	20.1	R	Cerebellar Lobule VIIIa (Hem)	2.8	24	−60	−50	6.8
	14.5	R	Cerebellar Lobule VIIIb (Hem)	2	N/A			N/A
	12	R	Cerebellar Lobule VIIa Crus II (Hem)	0.8	34	−62	−50	5.06
	4.8	R	Cerebellar Lobule VIIb (Hem)	0.7	N/A			N/A
57	N/A	R	Middle Frontal Gyrus	N/A	44	58	8	4.64
52	N/A	L	Middle Frontal Gyrus	N/A	−36	54	30	4.89
12	5.4	L	Hipp (CA)	0.7	−34	−26	−10	4.01
10	N/A	L	Middle Orbital Gyrus		−26	56	−14	4.33
**EMGpm>0**								
17151	1403.1	R	Cerebellar Lobule VI (Hem)	73	6	−64	−18	14.74
	1395.2	L	Cerebellar Lobule VI (Hem)	69	−28	−60	−30	11.55
	606.6	R	Cerebellar Lobule VIIa Crus I (Hem)	18	N\A			N/A
	587.6	L	Cerebellar Lobule VIIa Crus I (Hem)	18.5	N\A			N/A
	533.3	R	Cerebellar Lobule V	64.2	12	−54	−22	11.15
	429	L	Cerebellar Lobule V	56.2	N\A			N/A
	336.7	R	hOC3v (V3v)	48.2	N\A			N/A
	263.8	R	hOC4v (V4)	47.4	N\A			N/A
	232.9	R	Cerebellar Lobule VI (Vermis)	96.7	N\A			N/A
	224	L	Cerebellar Lobules I–IV (Hem)	44.4	N\A			N/A
	214.8	L	Cerebellar Lobule VI (Vermis)	99.5	N\A			N/A
	196.4	R	Area 18	11.8	N\A			N/A
	178.4	L	hOC3v (V3v)	26.4	N\A			N/A
	N/A	L	Pallidum	N/A	−24	−8	2	8.68
	N/A	L/R	Insula, Thalamus, Putamen	N/A	N\A			N/A
	N/A	R	Pallidum	N/A	N\A			N/A
11038	1995.3	L	Area 6 (SMA)	46.6	−24	−4	62	8.04
	1857.5	R	Area 6	43.5	N/A			N/A
	461.9	L	Area 2	51.3	N/A			N/A
	421	R	Area 2	44.3	38	−34	44	7.42
	197.1	L	IPC (PFt)	49	N/A			N/A
	184.4	R	IPC (PFt)	42.1	N/A			N/A
	172.6	L	hIP1	37.5	−30	−46	40	8.34
	169.5	R	Area 1	20.4	54	−30	56	7.02
	138.9	L	IPC (PF)	14.1	N/A			N/A
	138.3	L	Area 4p	24.4	N/A			N/A
	121.5	L	hIP2	53.8	N/A			N/A
	121.4	L	hIP3	43.3	N/A			N/A
	114.6	L	Area 4a	9.9	N/A			N/A
	N/A	R	Middle Cingulate Cortex	N/A	12	4	44	8.22
	N/A	R	hIP1,hIP2,hIP3, BA3b, BA4a, BA4p	N/A	N\A			N/A
	N/A	L	BA3a, BA3b	N/A	N\A			N/A
434	332.4	L	SPL (7A)	20.1	−16	−68	50	5.9
	52.3	L	SPL (7P)	9.6	−14	−70	58	5.51
	12	L	SPL (5L)	2.2	N\A			N/A
427	150.5	R	SPL (7A)	13.9	26	−54	56	4.24
	75.1	R	SPL (7P)	11.2	14	−68	56	4.49
	39.9	R	SPL (7PC)	9.8	34	−54	62	4.81
	34.6	R	hIP3	11.3	N\A			N/A
178	144.9	L	Area 44	12.4	−56	10	14	4.94
	6.1	L	Area 6	0.1	−58	8	34	4.19
152	N/A	R	Middle Frontal Gyrus		40	50	28	5.63
111	N/A	R	Middle Frontal Gyrus	N/A	48	52	4	4.86
92	1.5	L	Area 44	0.1	N\A			N/A
		L	Temporal Pole	N/A	−58	12	−4	5.13
20	N/A	L	Middle Orbital Gyrus	N/A	−26	56	−14	4.9
**PPI>0**								
3064	990	L	Area 6 (SMA)	23.1	−4	−22	50	9.88
	334.6	L	Area 2	37.2	−58	−22	40	8.21
	334.5	R	Area 6	7.8	N\A			N/A
	181.6	L	Area 3b	28.3	N\A			N/A
	177.3	L	Area 1	19.2	−32	−46	56	7.07
	174	L	Area 4a	15	−58	−16	42	6.95
	97.3	L	Area 4p	17.1	N\A			N/A
	75.9	L	IPC (PFt)	18.9	N\A			N/A
	31.4	L	Area 3a	6.3	N\A			N/A
	N/A	L	Middle Cingulate Cortex	N/A	−8	−24	48	10.08
		L	SPL(7PC)	N/A	N\A			N/A
1671	603.9	R	Cerebellar Lobule VI (Hem)	32.7	22	−50	−22	10.62
	170	R	hOC4v (V4)	31.8	N\A			N/A
	51.8	R	hOC5 (V5)	52.1	54	−68	0	6.99
	45	R	hOC3v (V3v)	6.7	N\A			N/A
	33.3	R	Cerebellar Lobule V	4.2	N\A			N/A
	22	R	Cerebellar Lobule VIIa Crus I (Hem)	0.7	N\A			N/A
	N/A	R	Fusiform Gyrus	N/A	32	−58	−14	7.42
	N/A	R	Middle Temporal Gyrus	N/A	56	−70	2	7.17
	N/A	R	Inferior Temporal Gyrus	N/A	52	−68	−6	5.39
1327	389.1	L	Cerebellar Lobule VI (Hem)	20	−28	−64	−22	9.15
	147.3	L	Cerebellar Lobule VIIa Crus I (Hem)	4.8	−36	−60	−30	6.24
	81.5	L	hOC4v (V4)	11.7	N\A			N/A
	46	L	hOC5 (V5)	63.4	N\A			N/A
	15.8	L	hOC3v (V3v)	2.4	N\A			N/A
	N/A	L	Middle Temporal Gyrus	N/A	−44	−70	8	7.2
	N/A	L	Fusiform Gyrus	N/A	−32	−60	−16	7.18
	N/A	L	Cerebellum	N/A	−32	−72	−20	5.57
	N/A	L	Inferior Occipital Gyrus	N/A	−38	−72	−10	5.49
	N/A	L	Middle Occipital Gyrus	N/A	−50	−70	−2	5.32
646	214.6	R	Area 1	25.9	58	−12	38	8.09
	130.8	R	Area 3b	14.2	62	−14	28	4.84
	100.6	R	Area 2	10.6	46	−26	52	6.58
	49.4	R	IPC (PFt)	11.3	54	−26	46	4.18
	39.5	R	Area 6	0.9	N\A			N/A
	11.1	R	Area 4a	1	N\A			N/A
	6.9	R	IPC (PFop)	2.5	54	−20	36	8.69
	N/A	R	Precentral Gyrus	N/A	60	−10	48	5.76
	N/A	R	Postcentral Gyrus	N/A	66	−12	38	5.63
	N/A	R	SupraMarginal Gyrus	N/A	68	−16	30	5.48
556	365	R	Area 44	41.6	62	14	26	7.5
	37.4	R	Area 45	3.5	N\A			N/A
	N/A	R	Rolandic Operculum	N/A	48	4	6	5.66
	N/A	R	Temporal Pole	N/A	62	10	−2	4.85
436	103.6	L	Area 44	8.9	−48	10	4	6.04
	N/A	L	Superior Temporal Gyrus	N/A	−52	6	−4	6.42
403	220.8	L	OP 1	37.1	−52	−32	24	7.35
	95.9	L	IPC (PFcm)	25.5	−48	−32	20	7.92
	49.6	L	IPC (PFop)	17.5	N\A			N/A
	7.1	L	IPC (PF)	0.7	N\A			N/A
	5.3	L	OP 4	0.9	N\A			N/A
	N/A	L	Superior Temporal Gyrus	N/A	−54	−40	20	4.66
361	138.3	R	IPC (PF)	15.6	64	−36	12	10.05
	57.6	R	OP 1	11.3	66	−20	14	5.26
	33.9	R	IPC (PFcm)	10.7	N\A			N/A
204	N/A	L	Thalamus	N/A	−8	−22	4	7.02
143	31.9	L	Amyg (SF)	17	−24	−2	−10	6.11
	N/A	L	Putamen	N/A	−22	8	−4	5.57
85	N/A	L	Putamen	N/A	−22	8	10	5.66
81	N/A	R	Putamen	N/A	24	10	−4	6.53
47	N/A	R	Insula	N/A	30	22	10	5.29
44	N/A	R	Thalamus	N/A	6	−24	−2	5.43
18	17.9	L	Area 6	0.4	−52	−4	44	4.58
15	N/A	R	Middle Cingulate Cortex	N/A	10	20	30	4.48
14	4.5	L	Area 18	0.3	−8	−62	−2	4.37
	N/A	L	Cerebellar Vermis	N/A	−4	−70	−6	4.61
12	5.4	R	Hipp (SUB)	1	16	−40	−4	4.91
	N/A	R	Lingual Gyrus	N/A	12	−42	−2	5.21
11	1.6	L	Area 17	0.1	N\A			N/A
	N/A	L	Lingual Gyrus	N/A	−24	−62	−6	4.73

From left to right we first list the cluster size in number of voxels. Then if the cluster encompasses cytoarchitectonically mapped brain regions (CytoArea, as by the Anatomy toolbox), the number of voxels activated within that CytoArea; hemisphere; name of CytoArea and the percentage of that CytoArea activated within this cluster. If the cluster extends beyond CytoAreas, the macroanatomical name are indicated instead, but the number of voxels within the CytoArea and the % activated are then not available (N/A). The final two columns apply if a local maximum falls within the Cyto- or anatomical area, in which case we mention the MNI coordinates (in mm) and the T value of the maximum. Note that if an area encompasses less than 1% of the cluster, the anatomy toolbox does not provide the Number of voxels or % of CytoArea activated, but we still list these clusters here for completeness because they encompass more than our threshold of 10 voxels. For the entire table, the voxelwise threshold was punc<0.001, all clusters had at least 10 voxels and also survived qfdr<0.05.

The inverse contrasts ACT<PASS and EMGpm<0 mainly recruited areas along the superior temporal sulcus, parietal operculum and cingulate cortex (Table S1 in File S1). In line with our results, reduction of tactile responses in these areas have been previously described in humans [15] (anterior cingulate cortex and parietal operculum) and monkeys [19] (superior temporal sulcus) while participants were actively generating the tactile stimulus. In the interest of our focus on BA2, the results in ACT<PASS and EMGpm<0 will not be further discussed.

As expected given that the EMG was higher in ACT than PASS, the comparisons between ACT and PASS and EMGpm versus zero showed very similar activations. Conceptually, if the EMG is taken as a proxy for motor efference, and thus efference-copy, EMGpm>0 is the most direct localization of the efference-copy effect as itcan capture variance even within conditions, and will thus be used instead of ACT>PASS throughout the remainder of the paper.

### Psycho-Physiological Interaction (PPI)

Increased activation of BA2 during blocks with higher EMG activity could be due to increased re-afference (i.e. more somatosensory input from the active hand) or efference-copy (more input from motor programming regions). If signals from motor regions contribute to the heightened BA2 activity during blocks with greater muscle activity, then the correlation between BA2 and motor regions should be higher on blocks with high muscle activity (i.e., active blocks) than on blocks with low muscle activity (i.e., passive blocks), when little efference-copy signals should be sent. Therefore, we performed a PPI interaction analysis with BA2 as the seed region (Fig. S1 in File S1) and EMG as the interacting physiological signal to find areas where the connection with BA2 increases on blocks with high muscle activity.

The results are presented in Figure 1g and Table 1. Supporting the influence of motor signals on SI, we found a large cluster with peaks in the SMA, which shows higher connectivity with SI during trials with more EMG, and hence, motor command generation [13]. A number of other regions associated with motor control also showed increased connectivity: PM, PF, M1, and cerebellum, in accord with the results found using ischemia [14]. However, there was also a peak in bilateral BA3b, which suggests an alternative explanation of why BA2 activity is heightened in blocks with high EMG. Proprioceptive and tactile feedback was similar but not identical during ACT and PASS blocks, so it is possible that heightened BA2 activity on blocks with high EMG could be due to the differences in somatosensory re-afference from BA3b to BA2 through what we will call the ‘body-loop’.

No voxels surviving FDR correction were found for the inverse, negative correlation (the first 16 voxels cluster within the gray matter appears at p*_unc_*<0.002, q*_FDR_*>0.99, in the left hippocampus at MNI -30 -32 -12).

### Partial Correlations

**Figure 2:**
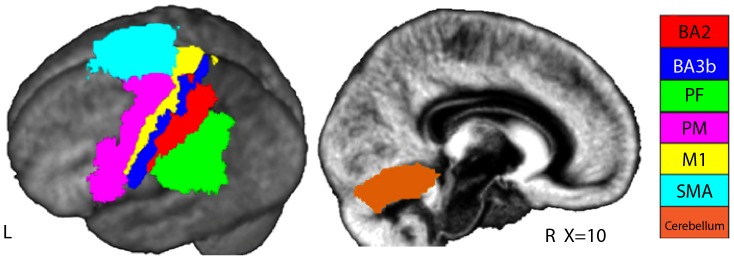
Regions of interest used in the partial correlation analysis. As mentioned in the text, left BA2 and left BA3b maps were directly selected from the toolbox; left PF included the PF, PFt, PFop, PFm, PFcm; left M1 included the BA4a and 4p; bilateral SMA was obtained by intersecting (Marsbar, http://marsbar.sourceforge.net) left and right BA6 maps with a box containing all voxels along y and z, but only from −17 to +17 along x; left PM resulted from the combination of BA6 and 44 and used all x<−17; Cerebellum included right lobule 5 and 6.

To explore whether the modulation of BA2 by regions involved in motor programming could simply be due to re-afference through the body-loop, we calculated partial correlations between activity in BA2 and the candidate motor control regions (SMA, PM, M1, PF and cerebellum) (Fig. 2). These partial correlations were obtained, in different analyses, after removing the variance shared (i) with the generic task time course (after HRF convolution), to remove variance due to the timing of the squeezing task; or (ii) the generic task and BA3b time courses, to exclude variance that could be associated with re-afference through BA3b. Figure S2 in File S1 illustrates the rationale behind removing the generic task time-course (after HRF convolution), and calculating partial correlations over the entire (residual) time course of a run. If a ROI responds similarly to ACT and PASS trials, regressing out the generic task time course will generate an essentially flat residual, with only noise left. If the ROI responds differently to ACT and PASS trials, regressing out the generic task will preserve the variance between ACT and PASS trials in the residuals. Performing a correlation between the residuals across ROIs then specifically looks at whether variance in responses between ACT and PASS trials in one ROI predicts variance in the other, as would be expected if efference-copy signals are transmitted along that path. However, the entire time-course of each ROI flows into the analysis, so that spontaneous (resting-state-like) fluctuations in one region would also remain in the residual time-course, and its transmission along the path would also benefit the analysis.

**Figure 3:**
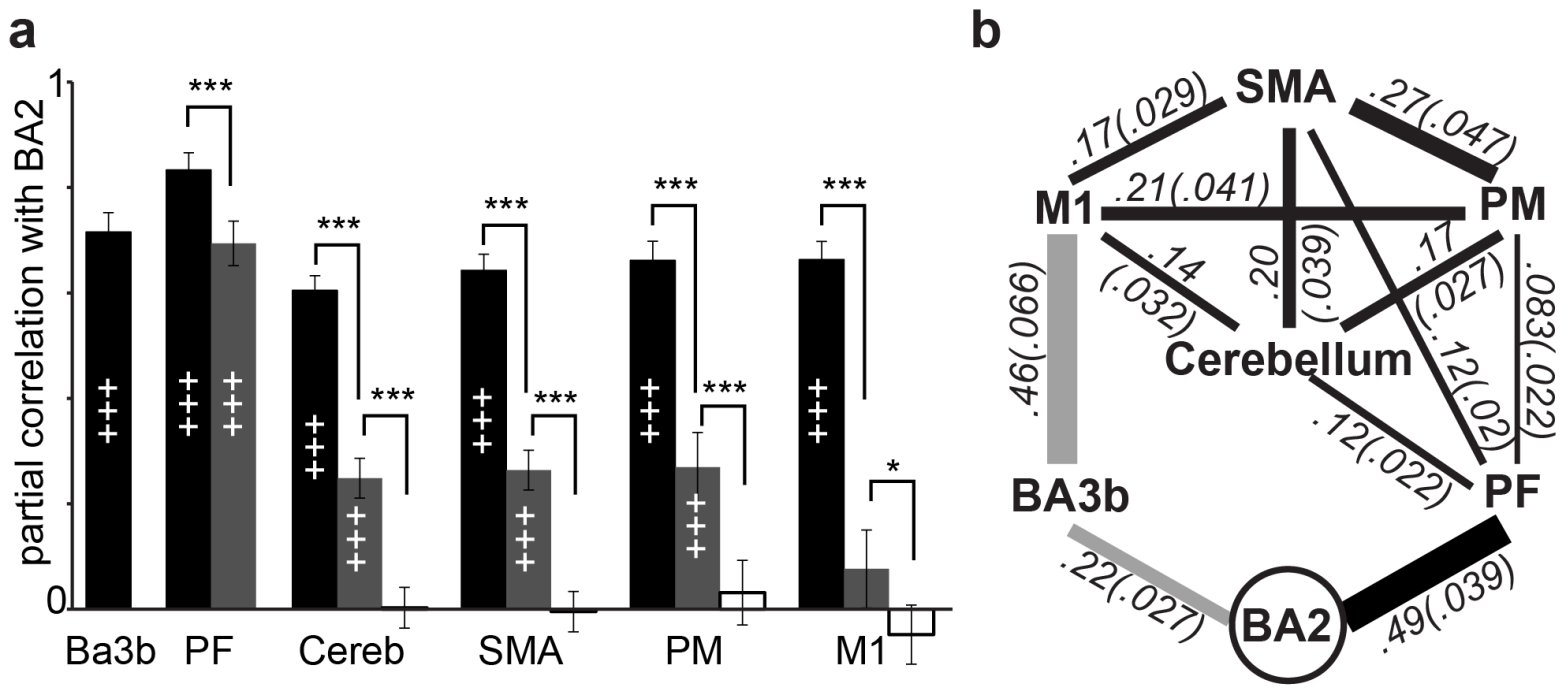
Partial Correlation and ICOV analysis results. (**a**) Partial correlation between BA2 and the key ROIs revealed by the PPI analysis as a function of the variance that has been removed (task only, black bars; task and BA3b, gray bars; task and BA3b and PF, white bars). ***: one tailed paired t-test p<0.001 Bonferroni-corrected for 5 ROIs (task only vs. task and BA3b removed) or 4 ROIs (task and BA3b vs. task, BA3b and PF). *: same at p<0.05. +++: one-tailed t-test against zero, p<0.001, Bonferroni-corrected for 6 ROIs (task only), 5 ROIs (task and BA3b) or 4 ROIs (task and BA3b and PF). (**b**) ICOV analysis revealing the pattern of direct connectivity between the selected ROIs. Connection strength (visually presented as line thickness), as average partial correlation value (± standard deviation), is indicated on each significant partial correlation. Connections between the nodes represent all the significant partial correlations (at p<0.05, bonferroni corrected for 21 possible pair-wise correlations, two-tailed t-test). Connections in grey are likely to represent re-afference through the body loop, those in black neural connections that could carry efference-copy signals.

Correlations that only partial out the task (Fig. 3a, black bars) confirm the significant link between BA2 and all the motor control regions as well as BA3b. Removing the variance shared with BA3b (gray bars) reduces the correlation with M1 to non-significance (p>0.8 after *b*onferroni *c*orrection, *b.c.*, for 5 ROI), suggesting that the association between M1 and BA2 could be entirely mediated by the body-loop, i.e. by BA3b. For PF, cerebellum, SMA and PM, the correlation with BA2 is reduced (matched-sample t-test, all p<0.001 after *b.c.* for 5 ROI) but remains significant (all p<0.003 after *b.c.* for 5 ROIs). This suggests that these regions are linked to BA2 both through the body-loop and through an efference-copy. Finally, because PF shows a particularly high partial correlation with BA2 after removing BA3b variance, and because PF is a key anatomical hub linking frontal motor regions with BA2 [20], [21] we explored if PF mediates the effect of SMA, PM and cerebellum on BA2, by additionally removing variance shared with PF (i.e., a partial correlation calculated after removing the variance shared with the generic task, BA3b and PF time courses; white bars). Doing so significantly reduced the partial correlations for all the ROIs (for SMA, PM and cerebellum, p<0.001; for M1, p<0.03 after *b.c.* for 4 ROIs), and all partial correlations were no longer significantly above zero (all p>0.2 even without *b.c.* for 4 ROIs), confirming a likely mediation by PF.

### Inverse covariance method

For a more comprehensive path analysis, we used the inverse covariance method, that identifies which nodes have direct connections by exploring the significance of the partial correlation between these regions after removing variance shared with any other ROIs or the task (see methods). This analysis revealed two pathways through which BA2 is connected with motor structures: one through BA3b (Fig. 3b, gray lines) and one through PF (black lines).

## Discussion

In our study, we challenge the validity of reverse inferences, suggesting that activations in BA2 exclusively reflect somatosensory processes, by investigating whether BA2 activation can instead also reflect motor commands (e.g. efference-copies), as suggested by modern theories of motor control [8]–[12], [22] We varied the efference-copy signal by making participants squeeze a soft material in their hand either actively or passively. We measured the EMG activity in the participants' lower arm to quantify the amount of motor efference. We then used the magnitude of this measure on a given trial as a proxy for the magnitude of the efference-copy.

By correlating the EMG with the BOLD signals throughout the brain we show that in addition to early somatosensory regions (BA3b) and regions involved in motor programming (SMA, PM, M1, cerebellum and PF), BA2 activity was also positively correlated with the EMG signal. This correlation is compatible with the *efference-copy account*: BA2 activity is higher on high EMG trials because higher activity in motor regions, SMA in particular [13] would lead to higher efference-copy signals to BA2 through the known anatomical connections between the motor structures and BA2 [20] in particular through area PF [21]. The presence of a similar correlation between EMG and BA3b is however compatible with an alternative *body-loop* account: despite our efforts to equate tactile sensations across conditions, the high EMG (active) trials might still have induced stronger tactile sensations that then activated BA2 more strongly via BA3b [20]. Because BA2 and BA3b are anatomically close and a 9 mm spatial smoothing was used in the preprocessing there is the possibility that overlapped voxels exist in these two regions. But this possibility would not weaken our conclusion because in the partial correlation, any smoothing overlap would express itself as a linear combination of signals, which would be taken out in the partial correlation. For example, the partial correlation of BA2 and PF, after removing BA3b, would only become smaller if we had more overlap in signal through smoothing. Therefore, the remaining significant correlation shown here stands.

A PPI analysis revealed that the connectivity with BA2 is augmented as a function of EMG with both somatosensory (BA3b) and motor control regions (SMA, PM, PM, M1, cerebellum and PF). This analysis is therefore again equally consistent with a body-loop (mediated by Ba3b) and efference-copy account of the BA2 modulation.

To establish whether some of the correlation in brain activity between BA2 and the motor control regions reflects an efference-copy, we removed any variance shared with BA3b using the most robust connectivity analyses available: partial correlations [23]. Results indicated that although part of the association between the activity in these motor control structures and BA2 seems indeed to be mediated by BA3b, for all regions except M1, another significant part is not. This shared variance between BA2 and the motor control regions, not mediated by BA3b, is exactly what efference-copy theories would predict, and makes it less likely that the inevitable tactile differences between the conditions could have been the only driving force behind the differential BA2 activity. A mathematically similar analysis, the inverse covariance method, corroborated this conclusion: BA2 is linked to motor control structures along two complementary paths that map onto the notion of a body-loop and an efference-copy. The body-loop corresponds to a path where motor control structures feed onto M1, which feeds onto BA3b and finally BA2. Because no direct anatomical connections exist between M1 and BA3b [24] this M1→BA3b pathway probably reflects M1 triggering body motion that changed tactile input to BA3b. The other pathway involves the motor control structures feeding onto PF then BA2. This pathway is in agreement with the main anatomical connections between frontal structures and BA2 [20], [21] and is therefore likely to reflect connections conveying an efference-copy.

While BA1 is known to play a critical role in relaying information from BA3b to BA2, this region is spatially so close to BA2 and BA3b, that its signal would have been highly correlated with that of the regions we already model. In the interest of the balance between accuracy and complexity BA1 was therefore not modeled.

Voluntary action is thought to originate in the frontal lobe, and the efference-copy could derive from premotor, supplementary motor and/or primary motor regions. Although most of the previous experiments are compatible with many of these routes, Haggard and colleagues identified SMA as a strong candidate [13]. Our own data indicates that SMA and/or PM, but not M1, are likely frontal source of the efference-copy to the somatosensory cortex, and suggest that PF is the main hub through which this efference-copy is sent to BA2. The cerebellum also seems to mediate part of that information in agreement with many theories [3], [9], [22].

Two families of methods currently exist to explore connectivity in fMRI data [23]. Undirected methods explore which brain regions are connected (directly or indirectly) using (partial) correlations, and simulations indicate these methods to be accurate and reliable [23]. Directed methods additionally attempt to derive the direction of information flow across regions but often lead to erroneous directions, and are thus less reliable [23], [25]. Also in our case, undirected, correlation based analyses lead to a stable patterns of connectivity while our attempts to use directional methods (Dynamic Causal Modeling, [26]) lead to less stable results. In particular, the connection pattern, complexity or number of ROIs included in the model comparison altered depending on whether the winning directed model explained BA2 activation differences in terms of efferenc-copy alone, a direct input to BA2 or as a combination of efference-copy and re-afference (see Supplementary Method S1, and Supplementary Fig. S3 and S4 all in File S1). Accordingly, we decided not to present or interpret the results of the directed analysis measures any further. With this caveat in mind, that frontal motor regions send the efference-copy to PF and then onwards to BA2 is one of the interpretations of the data. Alternatively PF might be the origin of the ‘decision’ to move, sending information to frontal motor regions to generate an overt movement and to BA2 as somatosensory predictions. Attributing a seminal role to the parietal lobe in the generation of visually instructed action is compatible with findings that electro-stimulation of the posterior parietal lobe can generate a volition to act [7]. Finally, undirected methods by themselves cannot exclude that it is BA2 that sends more somatosensory information to PF and frontal motor regions during the active than passive condition. The latter alternative is, however, rendered unlikely by evidence from an experiment using ischemia to reduce somatosensory re-afference [14]. In this experiment, ischemia reduced SI activation during passive ankle movements. If information exchange during active movements between premotor and SI regions were only to reflect somatosensory re-afference, this manipulation should have also reduced SI activation during active movements, which was not the case. Additional evidence that motor signals are sent from motor to somatosensory cortices stems from a study in rodents that found that while rodents palpate objects with their whiskers, the vibrissal motor cortex (vM1) sends motor information about whisker movements to the vibrissal somatosensory cortex (vS1) [27]. Electroencephalographic investigations might in the future provide data with higher temporal resolution to further disentangle these alternatives.

Generally, our data dovetail well with those of the study of Weber and colleagues [28] who recorded BA2 neurons in monkeys that showed changes in activity preceding active movements, of London and colleagues [29] who recorded neurons within SI (in particular BA2) that only discharged during passive and others only during active movements; and of Christensen and colleagues [14], who, by depriving the brain of the afferent input to SI, provided evidence for the presence of an efference-copy signal to BA2. By maintaining normal somatosensory afference in our experiment, but keeping it relatively constant across active and passive trials with very different levels of efference-copy signal, we provide evidence that even in the context of normal physiological afference, EMG-correlated neural signals from the SMA and/or PM have a significant predictive power on BA2 activation levels.

That early studies failed to find a difference in BA2 activity when comparing active and passive conditions could be due to a lack of power since they included only 6 participants [15], [16]. That one study measured a reduction in BA2 activation in active compared to passive finger-tapping [17] is however compatible with the idea that an efference-copy modulates BA2 activation but raises the question of when such an efference-copy augments and when it decreases BA2 activation.

Our study has a number of limitations that should be kept in mind. First, some residual motor activity was present even in the passive condition, and our data should not be seen as a contrast between conditions with and without motor command. To address this issue, we used statistics that explore trial-by-trial differences to explore if trials with more/less motor command show stronger/weaker activation in BA2 and more/less connectivity with motor control structures. Second, there are inevitable differences in the somatosensory consequences associated with the active and passive condition. We believe that such somatosensory differences are unlikely to account for the BA2 modulation we observed because the tactile input was actually stronger in the passive condition (in which the pressure of the hand of the experimenter was added to the counter-pressure of the material to be squeezed), whilst BA2 activation was higher in the active condition. We further tried to minimize the impact of such differences by excluding variance mediated by BA3b, as a proxy for somatosensory input from the body. However, our results should be examined with the caveat in mind, that we cannot entirely exclude the possibility that some afferent somatosensory signals may have been more intense in the active condition and may have bypassed BA3b. Finally, we use a number of methods (GLM, PPI, partial correlations), that all assume linear models in which different sources of influence on a region (BA2 in particular) add to each other. As in most BOLD-MRI studies, it should be noted, that this is only an approximation of how neural information is actually transformed into BOLD activity. Ultimately, these limitations will need to be overcome by converging evidence from different experiments investigating the influence of efference-copies to SI using different manipulations (ischemia, passive vs. active etc.) and different measurement techniques (BOLD fMRI, EEG etc.), each of which have different caveats.

In conclusion, our study suggests that the BOLD signal in BA2 can, under certain circumstances, reflect an input from motor control structures (SMA, PM, the cerebellum or PF in particular). This provides neural evidence for the recent view that efference-copy signals and internal models are part of the neural architecture of motor control [8]–[12], [22]. It additionally invites us to interpret activations in SI more carefully. That BOLD activation in BA2 can be significantly explained, in the sense of partial correlations, by signals from these motor control regions that scale with motor efference and that cannot be explained by BA3b activity, favors interpreting our effect in BA2 as at least partially motor rather than purely somatosensory. Theoretical models suggest that an internal model transforms the motor efference-copy into predicted somatosensory consequences [1]–[5]. This interpretation would warrant calling the modulation of BA2 we measured somatosensory-motor rather than strictly motor. Accordingly, together with the data of Christensen et al. [14], London et al [29] and Weber et al [28] and the modern visions of sensorimotor control [8]–[12], [22], our experiment suggests that we should interpret activations in BA2 in fMRI experiments as evidence for *somatosensory-motor* coding. Interpreting BA2 activations as evidence for somatosensory as opposed to, and qualitatively distinct from, motor coding, on the other hand, seems no longer appropriate.

## Method

### Subjects

Nineteen right-handed subjects (11 male, 21.6 years ±4.5 s.e.m. ranged 18–40 years) with no history of neurological disorders participated in the experiment. One was excluded from all analyses due to electromyography recording problems and one from the connectivity (incl. partial correlation) analyses because a stronger EMG response during passive compared to active blocks suggested poor understanding of task instructions. The research was approved by the Medical Ethical Committee of the University Medical Center Groningen (NL) and all subjects signed a written informed consent form.

### Experimental Design

Participants and the experimenter wore a thin latex glove on their right hand (Fig. 1a). On the palm side of the subject's glove, bubble wrap was attached as an object to squeeze. During PASS, participants were shown a sequence of four 1 s red circles of decreasing size (Fig. 1b). At the onset of each circle, author CF squeezed the bubble wrap, by acting upon the subject's right hand fingers. During ACT, the circles were green instead of red, and the participant gently squeezed the bubble wrap. Because subject's and experimenter's gloves were glued together, during ACT the experimenter could follow, with her fingers, the subject's movements, introducing a light pressure (i.e. an afference signal) similar to the one in PASS. Prior to scanning, (i) participants and author CF rehearsed to make the squeezing force and range of motion as similar as possible in both conditions to ensure that somatosensory feedback would be closely matched, and (ii) participants were trained, by EMG biofeedback, to keep the EMG as small as possible during PASS. The 20 ACT and 20 PASS blocks were presented in a pseudo-randomized order. A random duration (10–14 s) centered gray circle separated the blocks.

### Electromyography

Surface EMG monitored muscle activity from the flexor digitorum superficialis (FDS) muscle. A bipolar recording was made from two electrodes, placed longitudinally with respect to the muscle fibers above the FDS on the skin, close to the more superficially positioned flexor carpi radialis muscle, using the BrainAmp MR plus system (Brain Products GmbH, Munich, Germany). The electrode locations were determined by observing and palpating muscle contractions, using maximum voluntary contractions (as measured by the EMG) towards the specific pulling direction of the FDS. A reference electrode was placed on the right wrist, at the processus styloideus. All data were recorded at 5 kHz using the Brain Vision Recorder 1.03 software (Brain Products, Munich, Germany). BrainVision Analyzer 1.05 was used to correct the EMG data for MRI artifacts using the standard averaging and subtraction method [30], which has been validated for its use in EMG [31]. A 10 Hz high-pass filter was applied to remove movement artifacts [32]. The data were then rectified and down sampled to 250 Hz. As a consequence of the rectification, the information on EMG burst-frequency is enhanced, thereby recovering the low frequency (<10 Hz) EMG content [33]. *MRI acquisition and preprocessing:* Whole brain functional MRI images (EPIs) were acquired, with a Philips Intera 3T Quasar whole body scanner, using a a T2-weighted echo-planar sequence (39 interleaved, 3.5 mm axial slices, no gap; TR = 2000 ms; TE = 30 ms; flip angle = 80°; FOV = 224×224 mm; 64×64 matrix of 3.5 mm isotropic voxels), and were followed by a whole brain T1-weighted anatomical image (1×1×1 mm), parallel to the bicommissural plane. All EPIs were slice-time corrected and realigned to the subject's mean EPI. The normalization parameters from the segmentation of the mean-co-registered T1 images were then applied to all EPIs. Data were smoothed with a 9 mm isotropic FWHM Gaussian kernel (SPM8; http:www.fil.ion.ucl.ac.uk/spm/software/spm8).

An MR-compatible 32-channel BrainAmp system (Brain Products, Munich, Germany) was used to record EEG simultaneously to investigate the relationship between EEG mu-suppression and BOLD signal, as reported in [34]. For most subjects, there was a drop in BOLD signal intensity over the left parietal lobe, likely an artifact caused by the EEG cables. SPM8 therefore considered these voxels out of the brain. The following procedure was used: “(a) all 19 subjects' smoothed mean EPIs were averaged into a grand mean EPI; (b) this grand mean EPI was divided by each subject's smoothed mean EPI; (c) we then multiplied, for each subject separately, all the smoothed EPIs by the subject's correction map obtained in point (b)” [34] (http://www.nin.knaw.nl/Portals/0/Department/keysers/Arnstein%20SupplementaryFigures.pdf). Additionally, regression analyses found no significant relationship between the amount of attenuation within regions of interest in a participant and the connectivity measures derived from those regions in that participant (see Supplementary Method S2 in File S1).

### MRI data analyses

In both GLMs blocks in which the task was performed incorrectly were modeled separately with a boxcar predictor of no interest and then convolved with the HRF. To account for head movements we included 24 parameters (three translations, three rotations, their first temporal derivative, their quadratic, and these head motion parameters shifted forward by 1TR), as covariates of no interest, not convolved with the HRF.

### Psycho-Physiological-Interaction (PPI) analysis

Activity in left BA2, defined using the Anatomy Toolbox 1.7 maps ([35] http://www.fz-juelich.de/ime/spm_anatomy_toolbox) for SPM8, was the physiological predictor for the PPI analysis. At the first level, for each subject, we visualized EMGpm>0 at p*_unc_*<0.001, and extracted the first eigenvariate from a 6 mm sphere, centered on the individual's absolute maximum within the left BA2 masked results(Fig. S1 in File S1). The EMGpm of each participant's original GLM was the psychological variable. The SPM8 PPI function then determined the interaction term. We used the psycho-physiological option because the EMG measurement, like a psychological variable, does not lag behind the underlying neural process. The physio-physiological option, terminologically more appropriate, would instead have de-convolved the EMGpm signal. A new GLM was then created for each participant using these three predictors. The parameter estimates for the interaction term were brought to second level analysis, comparing it against zero using a t-test. Only 14 out of the 17 subjects had a maximum within left BA2 at p*_unc_*<0.001. In the main text and Figure 1g, for the PPI analysis, we therefore only show group results coming from them. A similar analysis, reducing the thresholds for the ROI definition to include all 17 participants led to virtually identical results (see Supplementary Methods S3 and Fig. S5 in File S1). Similar results were also obtained, when a ROI resulting from the masking of the EMGpm group results (*p*<.001 uncorrected, k>10) with the anatomical BA2 was used as a mask at the first level (see Supplementary Methods S4 and Fig. S6 in File S1).

### Statistical Threshold

All analyses were initially thresholded at *p_unc_*<0.001 (k>10) at the second, group level. To control the overall false discovery rate, we only report results that also survive a voxelwise q*_FDR_*<0.05.

### Partial Correlation Analyses

Based on the PPI results, for the partial correlation analyses, we defined (Anatomy Toolbox [36]) seven anatomical ROIs: BA2 [37], BA3b [38], [39], PF [40], [41], cerebellum [42], SMA, PM [43] and M1 [44]. All, but SMA and cerebellum, only included the left hemisphere (Fig. 2). BA2 and BA3b maps were directly selected from the toolbox; PF included the PF, PFt, PFop, PFm, PFcm; and M1 the BA4a and 4p. Based on visual inspection of the averaged anatomy of our group, and on the Harvard-Oxford cortical atlas (http://www.cma.mgh.harvard.edu/fsl_atlas.html), to obtain the SMA, we intersected (Marsbar, http://marsbar.sourceforge.net) left and right BA6 maps with a box containing all voxels along y and z, but only from −17 to +17 along x. For left PM, we combined BA6 and 44 and used all x<−17. Cerebellum included right lobule 5 and 6, which contain the main cerebellar hand representation and are connected with motor, parietal and somatosensory hand representations in the cortex [45]. For each ROI and participant, we extracted the first eigen-time-course from all voxels for which EMGpm>0 at p*_unc_*<0.05. Only 14 participants contained at least 5 significant voxels in all ROIs, and the analysis was restricted to them. The mean partial correlation values for the 14 participants in the three correlation analyses were assessed at second level using a one tailed t-test against zero. All significant t-test results were also significant when using non-parametric tests (Wilcoxon-Signe Rank or Mann-Whitney U).

### Inverse Covariance Method

To identify which ROIs are directly connected, we explored the significance of the partial correlation between these regions after removing variance shared with the other ROIs and the task. Assuming that the matrix of plausible connections between the ROIs is sparse, the inverse covariance method (“glasso” implementation in the R Statistical package) leverages the fact that a full set of partial correlations can be computed using the inverse of the covariance (ICOV) matrix [46]. Briefly, each variable ‘i’ (ROIs in this context) is represented as a general linear model (GLM) comprising of all other variables ‘j’, under the constraint that the sum of the absolute coefficients (Cij) of the individual regressors be less than a given constant tuning parameter P. If Cij and Cji is zero, then the ij entry of the inverse covariance matrix is zero [47]. This Lasso shrinkage method [48], [49] sets many of the entries in the partial correlation matrix to zero as a function of P. Note that if P is very large the contraint has no effect and a full inverse covariance matrix (and hence a full partial correlation matrix) is obtained. But a small positive P sets many of the partial correlation values to zero, while resulting in different fitting errors for the model. We present the results corresponding to P = 0.01 and the results remain robust against slight variations of this value. The tuning parameter has the effect of controlling the number of predictors in the GLM.

## Supporting Information

File S1 **Contains the following:** Table S1: Clusters of activity resulting from the contrasts PASS>ACT and EMGpm<0. Figure S1: Illustration of the BA2 region used in PPI analysis for all the 14 subjects. The green shows the 6 mm sphere centered in the peak. The red shows the anatomical region of BA2. And yellow are the overlaps as well as the region used in the PPI analysis. Figure S2: Illustration of the Partial Correlation Logic. (A) If a ROI has an actual BOLD response similar during ACT and PASS blocks, regressing out the time course of the generic task (after HRF convolution) leaves only noise in the residuals. (B) If a ROI responds differently to ACT and PASS, regressing out the same generic task retains the variance between conditions in the residual time-course. These residuals can then serve to track how differences between ACT and PASS are transmitted from ROI to ROI. The time-courses in this figure are not actual data, but simulated data to caricature the concept. Figure S3: Graphical illustration of the six models compared in DCM. The RFX Bayesian model comparison results is in the graph on the right. The numbers in the top left of each graph correspond to those in the x-axis of the chart. Figure S4: Graphical illustration of the models including M1 and PM compared in the DCM analysis. The RFX Bayesian model comparison results is in the graph on the bottom. The numbers in the right underside of each graph correspond to those in the x-axis of the chart. Note that the generic task is always included as modulator of both BA3b and BA2, as in the previous analysis. Figure S5: PPIs group results. Green color: second level PPI results currently presented in the manuscript (T<4.02 at punc<0.001, all survive qfdr<0.05). The eigen-vectors were extracted from a 6 mm sphere centered on the local maxima within the anatomical BA2 ROI. Eigen-vectors were extracted at the single subject level at punc<0.001 for 14 out of 17 subjects. Red color: second level PPI results for the entire group of 17 subjects (T>3.69, punc<0.001, all voxels also survive qfdr<0.05). As for green, the eigen-vectors were extracted from a 6 mm sphere centered on the local maxima within the anatomical BA2 ROI. Eigen-vectors were extracted at single subject level within that region from all voxels where a subject showed a correlation with EMG, at punc<0.001 threshold for 14 out of 17 subjects, and at punc<0.5 for the remaining 4. Yellow color: overlap between Red and Green. Figure S6: PPIs group results. Green color: second level PPI results currently presented in the manuscript (T<4.02 at punc<0.001, all survive qfdr<0.05). The eigen-vectors were extracted from a 6 mm sphere centered on the local maxima within the anatomical BA2 ROI. Eigen-vectors were extracted at the single subject level at punc<0.001 for 14 out of 17 subjects. Red color: second level PPI results for the entire group of 17 subjects (T>3.69, punc<0.001, all voxels also survive qfdr<0.05) using the method define above (supplementary method S4 in File S1). Yellow color: overlap between Red and Green. Supplementary Method S1: Dynamic causal modeling (DCM). Supplementary Method S2: Influence of the EEG artifact on the partial correlation analyses. Supplementary Method S3: PPI analysis including all 17 subjects. Supplementary Method S4: PPI results using group-level results to define ROIs.
(DOC)
