# Supplementary material for: Functional Magnetic Resonance Imaging Connectivity Analyses Reveal Efference-Copy to Primary Somatosensory Area, BA2
Source: PLoS One. 2014 Jan 8;9(1):e84367. doi: 10.1371/journal.pone.0084367 (PMC3885571; doi:10.1371/journal.pone.0084367)
Supplement: File S1 — Contains the following: Table S1: Clusters of activity resulting from the contrasts PASS>ACT and EMGpm<0. Figure S1: Illustration of the BA2 region used in PPI analysis for all the 14 subjects. The green shows the 6 mm sphere centered in the peak. The red shows the anatomical region of BA2. And yellow are the overlaps as well as the region used in the PPI analysis. Figure S2: Illustration of the Partial Correlation Logic. (A) If a ROI has an actual BOLD response similar during ACT and PASS blocks, regressing out the time course of the generic task (after HRF convolution) leaves only noise in the residuals. (B) If a ROI responds differently to ACT and PASS, regressing out the same generic task retains the variance between conditions in the residual time-course. These residuals can then serve to track how differences between ACT and PASS are transmitted from ROI to ROI. The time-courses in this figure are not actual data, but simulated data to caricature the concept. Figure S3: Graphical illustration of the six models compared in DCM. The RFX Bayesian model comparison results is in the graph on the right. The numbers in the top left of each graph correspond to those in the x-axis of the chart. Figure S4: Graphical illustration of the models including M1 and PM compared in the DCM analysis. The RFX Bayesian model comparison results is in the graph on the bottom. The numbers in the right underside of each graph correspond to those in the x-axis of the chart. Note that the generic task is always included as modulator of both BA3b and BA2, as in the previous analysis. Figure S5: PPIs group results. Green color: second level PPI results currently presented in the manuscript (T<4.02 at punc<0.001, all survive qfdr<0.05). The eigen-vectors were extracted from a 6 mm sphere centered on the local maxima within the anatomical BA2 ROI. Eigen-vectors were extracted at the single subject level at punc<0.001 for 14 out of 17 subjects. Red color: second level PPI results for the ent [file pone.0084367.s001.doc]

**Functional Magnetic Resonance Imaging Connectivity Analyses Reveal Efference-Copy to Primary Somatosensory Area, BA2.**

F Cui^1,2^, D Arnstein^1^, RM Thomas^2^, NM Maurits^3^, C Keysers^1,2^, V Gazzola^1,2,*^

### **Supplementary Tables**

**Table S1. Clusters of activity resulting from the contrasts PASS>ACT and EMGpm<0**

From left to right we first list the cluster size in number of voxels. Then if the cluster encompasses cytoarchitectonically mapped brain regions (CytoArea, as by the Anatomy toolbox), the number of voxels activated within that CytoArea; hemisphere; name of CytoArea and the percentage of that CytoArea activated within this cluster. If the cluster extends beyond CytoAreas, the macroanatomical name are indicated instead, but the number of voxels within the CytoArea and the % activated are then not available (N/A). The final two columns apply if a local maximum falls within the Cyto- or anatomical area, in which case we mention the MNI coordinates (in mm) and the T value of the maximum. Note that if an area encompasses less than 1% of the cluster, the anatomy toolbox does not provide the Number of voxels or % of CytoArea activated, but we still list these clusters here for completeness because they encompass more than our threshold of 10 voxels. For the entire table, the voxelwise threshold was p*_unc_*<0.001, all clusters had at least 10 voxels and also survived q*_fdr_*<0.05.

| **PASS>ACT** | | | | | | | | | | |
| --- | --- | --- | --- | --- | --- | --- | --- | --- | --- | --- |
| 1748 | 347 | R | IPC (PGa) | 36 | 60 | | -60 | | 26 | 7.6 |
|  | 321.1 | R | IPC (PGp) | 27.5 | 52 | | -68 | | 38 | 5.93 |
|  | 25.4 | R | hOC5 (V5) | 25.6 | N/A | | | | | N/A |
|  | N/A | R | Middle Temporal Gyrus | N/A | 56 | | -44 | | 10 | 6.72 |
| 1113 | N/A | R/L | Precuneus | N/A | 4 | | -54 | | 26 | 6.34 |
|  | N/A | R | Middle Cingulate Cortex | N/A | 4 | | -50 | | 32 | 6.23 |
|  | N/A | L | Cuneus | N/A | -14 | | -58 | | 26 | 4.3 |
| 1111 | N/A | R | Medial Temporal Pole | N/A | 40 | | 16 | | -32 | 7.52 |
|  | N/A | R | Middle Temporal Gyrus | N/A | 58 | | 4 | | -26 | 7.5 |
|  | N/A | R | Medial Temporal Pole | N/A | 48 | | 8 | | -30 | 5.73 |
|  | N/A | R | Superior Temporal Gyrus | N/A | 58 | | -8 | | -8 | 5.72 |
|  | N/A | R | Middle Temporal Gyrus | N/A | 66 | | -18 | | -10 | 5.62 |
| 959 | 515.4 | L | IPC (PGp) | 47.5 | -48 | | -76 | | 32 | 5.56 |
|  | 92.1 | L | IPC (PGa) | 11.9 | -48 | | -56 | | 24 | 4.5 |
|  | N/A | L | Middle Temporal Gyrus | N/A | -50 | | -62 | | 14 | 5.67 |
|  | N/A | L | Angular Gyrus | N/A | -44 | | -58 | | 26 | 4.58 |
| 917 | N/A | R/L | Mid Orbital Gyrus | N/A | 8 | | 52 | | -10 | 6.73 |
|  | N/A | L | Anterior Cingulate Cortex | N/A | -2 | | 38 | | -8 | 4.45 |
|  | N/A | L | Mid Orbital Gyrus | N/A | -4 | | 42 | | -12 | 4.37 |
|  | N/A | R/L | Anterior Cingulate Cortex | N/A | 6 | | 40 | | 0 | 4.24 |
| 285 | N/A | L | Middle Temporal Gyrus | N/A | -54 | | -36 | | 0 | 5.27 |
| 228 | N/A | L | Middle Temporal Gyrus | N/A | -54 | | 4 | | -24 | 6.79 |
|  | N/A | L | Medial Temporal Pole | N/A | -44 | | 14 | | -30 | 4.71 |
| 227 | N/A | L | Middle Frontal Gyrus | N/A | -32 | | 28 | | 54 | 4.98 |
| 196 | N/A | R/L | Superior Medial Gyrus | N/A | 6 | | 56 | | 18 | 4.68 |
|  | N/A | R | Anterior Cingulate Cortex | N/A | 8 | | 54 | | 10 | 3.77 |
| 93 | N/A | L | Inferior Frontal Gyrus (p. Orbitalis) | N/A | -38 | | 36 | | -10 | 6.55 |
| 93 | 52.1 | R | Hipp (SUB) | 10.1 | N/A | | | | | N/A |
|  | 32.8 | R | Hipp (CA) | 4.1 | 26 | | -16 | | -20 | 7.33 |
|  | 3.1 | R | Hipp (FD) | 3.7 | N/A | | | | | N/A |
|  | 2.6 | R | Hipp (EC) | 0.4 | N/A | | | | | N/A |
| 50 | 21 | L | IPC (PFcm) | 5.6 | -44 | | -34 | | 16 | 4.53 |
|  | 16.8 | L | OP 1 | 2.8 | -38 | | -32 | | 18 | 4.68 |
|  | 6.4 | L | TE 1.1 | 3.8 | N/A | | | | | N/A |
|  | 1.9 | L | OP 2 | 1.6 | N/A | | | | | N/A |
| 30 | N/A | R | Middle Orbital Gyrus | N/A | 28 | | 38 | | -12 | 3.98 |
|  | N/A | R | Inferior Frontal Gyrus (p. Orbitalis) | N/A | 30 | | 36 | | -14 | 3.92 |
| 21 | N/A | R | Superior Frontal Gyrus | N/A | 24 | | 42 | | 52 | 4.12 |
| 21 | N/A | R | Superior Medial Gyrus | N/A | 8 | | 46 | | 44 | 4.44 |
| 19 | N/A | R | Superior Frontal Gyrus | N/A | 28 | | 56 | | 8 | 4.11 |
|  | N/A | R | Middle Frontal Gyrus | N/A | 26 | | 56 | | 2 | 3.77 |
| 16 | 16 | L | Cerebellar Lobule VIIa Crus II (Hem) | 1 | -16 | | -86 | | -38 | 4.45 |
| **EMGpm < 0** | | | | | | | | | | |
| 878 | N/A | R | Medial Temporal Pole | N/A | 40 | 16 | | -32 | | 9.8 |
|  | N/A | R | Middle Temporal Gyrus | N/A | 58 | -2 | | -20 | | 7.04 |
|  | N/A | R | Superior Temporal Gyrus | N/A | 58 | -8 | | -10 | | 4.96 |
| 828 | N/A | R | Precuneus | N/A | 4 | -54 | | 26 | | 5.62 |
|  | N/A | R | Middle Cingulate Cortex | N/A | 4 | -54 | | 32 | | 5.61 |
|  | N/A | L/R | Precuneus | N/A | 0 | -56 | | 26 | | 5.42 |
| 692 | N/A | R/L | Mid Orbital Gyrus | N/A | 6 | 56 | | -10 | | 7.19 |
|  | N/A | L | Anterior Cingulate Cortex | N/A | -2 | 38 | | -8 | | 3.91 |
| 544 | 357.5 | L | IPC (PGp) | 32.9 | -48 | -76 | | 32 | | 5.4 |
|  | 63.9 | L | IPC (PGa) | 8.2 | N\A | | | | | N/A |
|  | N/A | L | Angular Gyrus | N/A | -48 | -56 | | 24 | | 4.58 |
|  | N/A | L | Middle Temporal Gyrus | N/A | -46 | -62 | | 12 | | 4.44 |
| 442 | 128.3 | R | IPC (PGa) | 13.3 | 62 | -58 | | 26 | | 5.32 |
|  | 123.9 | R | IPC (PGp) | 10.6 | 52 | -68 | | 38 | | 4.44 |
|  | N/A | R | Angular Gyrus | N/A | 62 | -58 | | 26 | | 5.32 |
|  | N/A | R | Middle Temporal Gyrus | N/A | 50 | -58 | | 8 | | 4.4 |
|  | N/A | R | Superior Temporal Gyrus | N/A | 54 | -32 | | 2 | | 4.1 |
|  | N/A | R | Middle Occipital Gyrus | N/A | 54 | -72 | | 26 | | 4.05 |
|  | N/A | R | Middle Temporal Gyrus | N/A | 52 | -54 | | 16 | | 3.94 |
| 190 | N/A | L/R | Superior Medial Gyrus | N/A | 10 | 60 | | 22 | | 4.49 |
| 187 | N/A | L | Middle Temporal Gyrus | N/A | -54 | 6 | | -24 | | 5.23 |
|  | N/A | L | Medial Temporal Pole | N/A | -44 | 14 | | -32 | | 4.93 |
|  | N/A | L | Middle Temporal Gyrus | N/A | -56 | 8 | | -28 | | 4.75 |
| 63 | N/A | L | Middle Frontal Gyrus | N/A | -30 | 22 | | 44 | | 4.22 |
| 45 | 27.6 | L | Area 3b | 4.3 | -58 | -8 | | 32 | | 4.46 |
|  | 9.3 | L | Area 4p | 1.6 | N\A | | | | | N/A |
|  | 4.9 | L | Area 3a | 1 | N\A | | | | | N/A |
|  | 3 | L | Area 1 | 0.3 | -62 | -6 | | 32 | | 3.73 |
| 34 | 16.8 | R | Area 3a | 3.6 | 54 | -6 | | 22 | | 4.51 |
|  | 12.1 | R | Area 3b | 1.3 | 56 | -6 | | 28 | | 4.31 |
|  | 4.4 | R | Area 4p | 0.9 | N\A | | | | | N/A |
| 26 | 12.6 |  | IPC (PFcm) | 3.4 | -44 | -34 | | 18 | | 4.8 |
|  | 11.4 |  | OP 1 | 1.9 | -40 | -32 | | 22 | | 4.44 |
|  | 1 |  | TE 1.1 | 0.6 | N\A | | | | | N/A |
| 12 | N/A | R | Superior Medial Gyrus | N/A | 8 | 46 | | 44 | | 4.26 |
| 12 | N/A | R | Superior Frontal Gyrus |  | 24 | 54 | | 8 | | 4.32 |

### **Supplementary Tables**


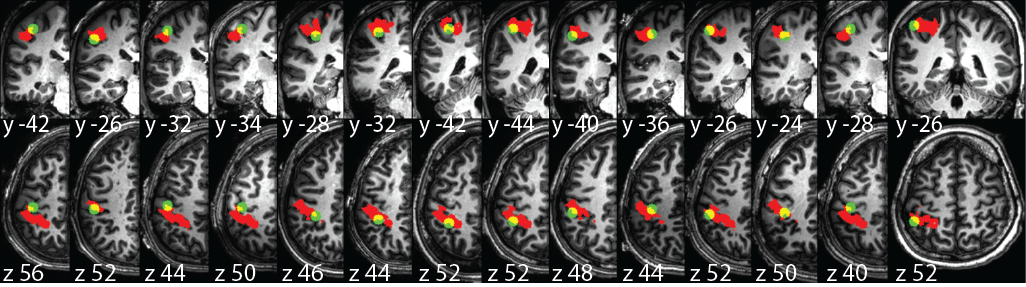


**Figure S1: Illustration of the BA2 region used in PPI analysis for all the 14 subjects.** The green shows the 6mm sphere centered in the peak. The red shows the anatomical region of BA2. And yellow are the overlaps as well as the region used in the PPI analysis.

# *
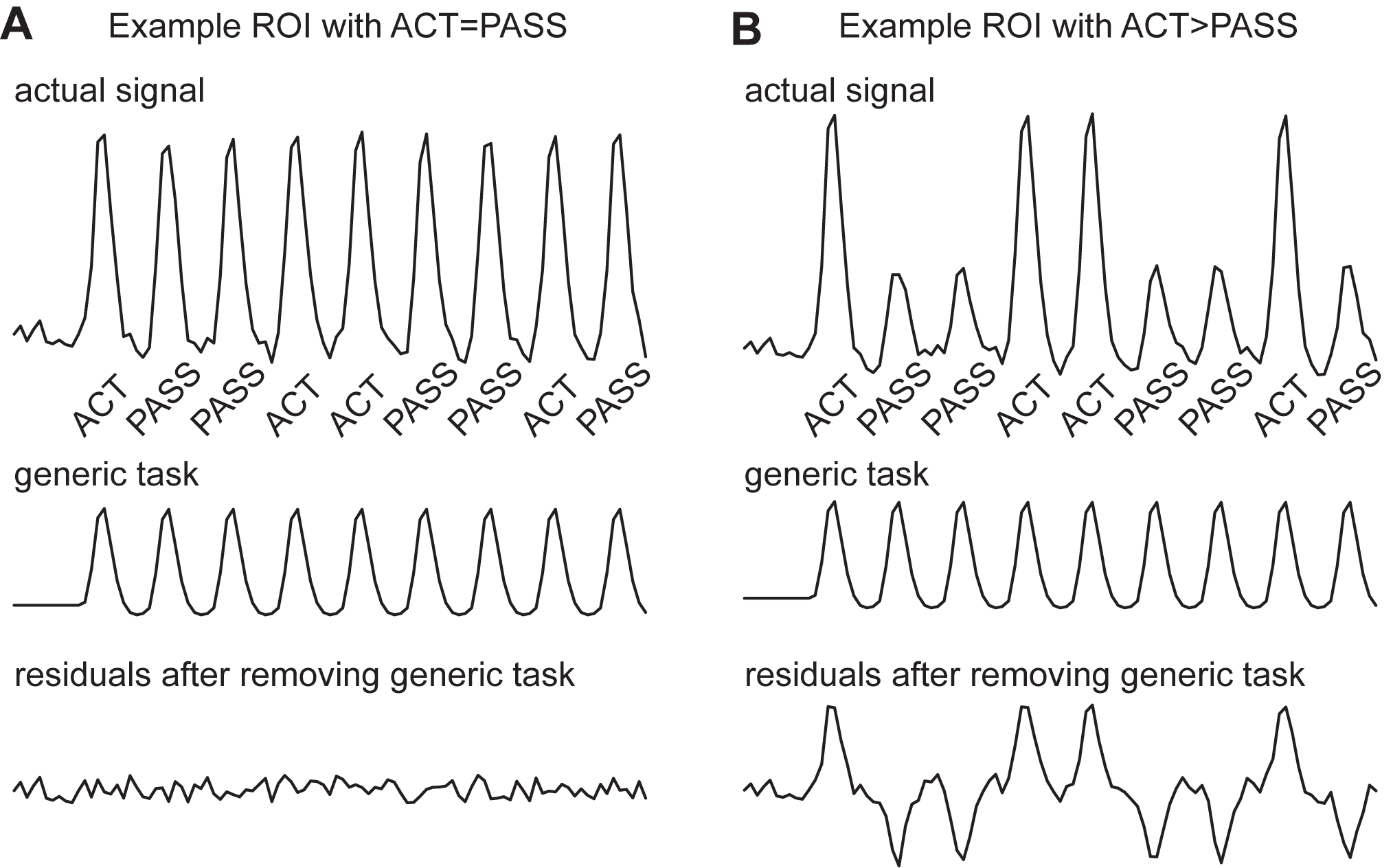
*

## Figure S2: Illustration of the Partial Correlation Logic. (A) If a ROI has an actual BOLD response similar during ACT and PASS blocks, regressing out the time course of the generic task (after HRF convolution) leaves only noise in the residuals. (B) If a ROI responds differently to ACT and PASS, regressing out the same generic task retains the variance between conditions in the residual time-course. These residuals can then serve to track how differences between ACT and PASS are transmitted from ROI to ROI. The time-courses in this figure are not actual data, but simulated data to caricature the concept.


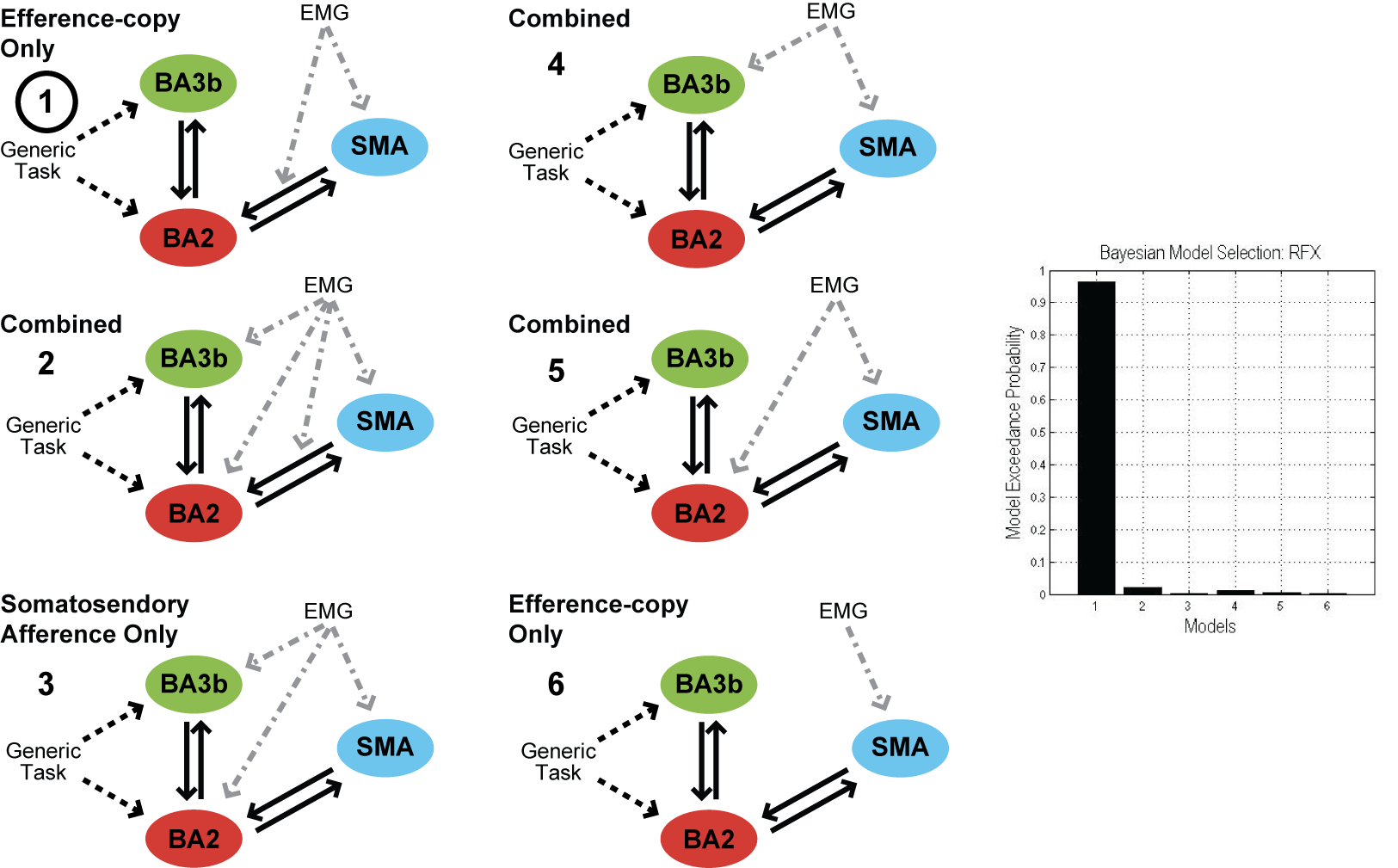


**Figure S3**: **Graphical illustration of the six models compared in DCM**. The RFX Bayesian model comparison results is in the graph on the right. The numbers in the top left of each graph correspond to those in the x-axis of the chart.


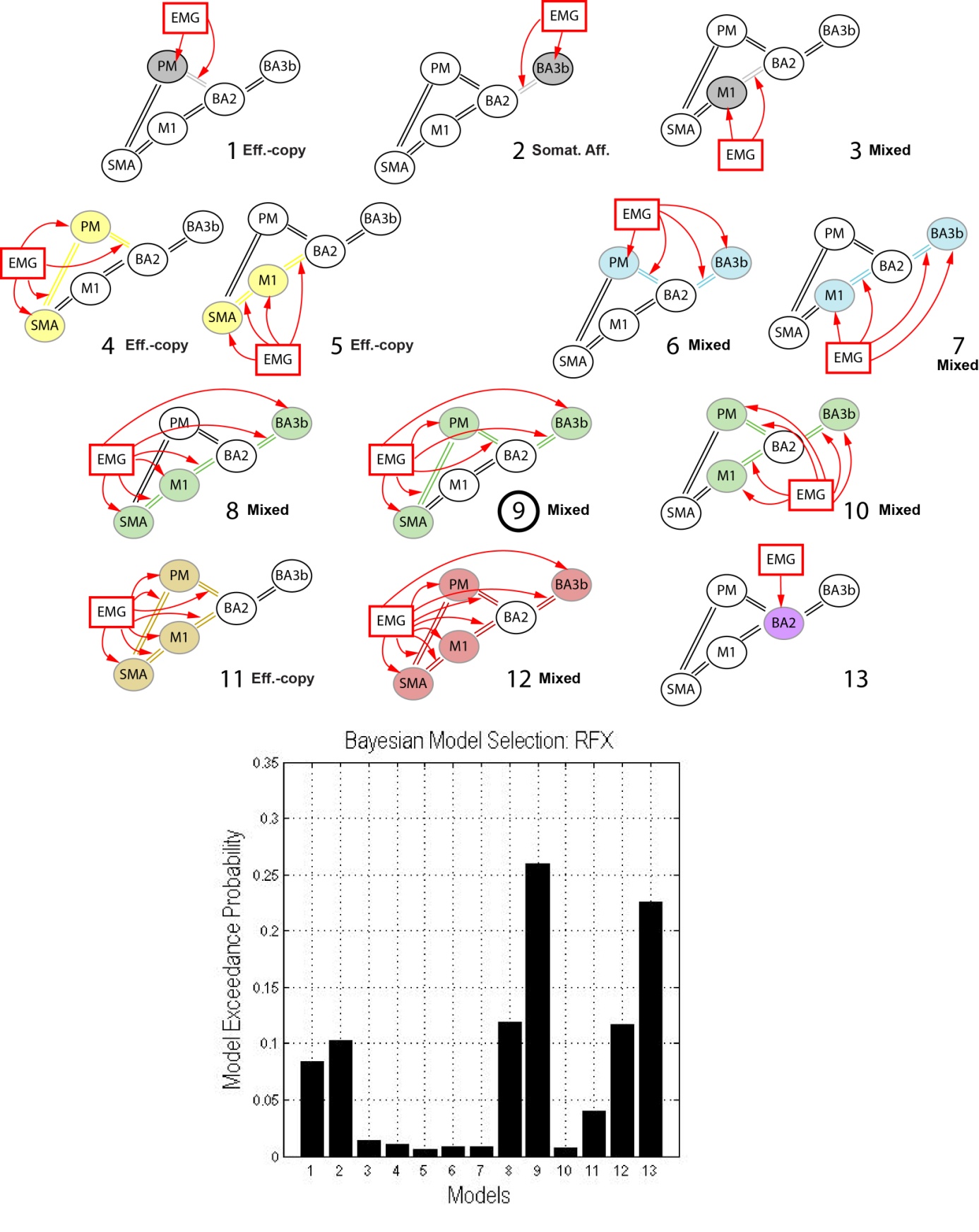


**Figure S4**: **Graphical illustration of the models including M1 and PM compared in the DCM analysis.** The RFX Bayesian model comparison results is in the graph on the bottom. The numbers in the right underside of each graph correspond to those in the x-axis of the chart. Note that the generic task is always included as modulator of both BA3b and BA2, as in the previous analysis.


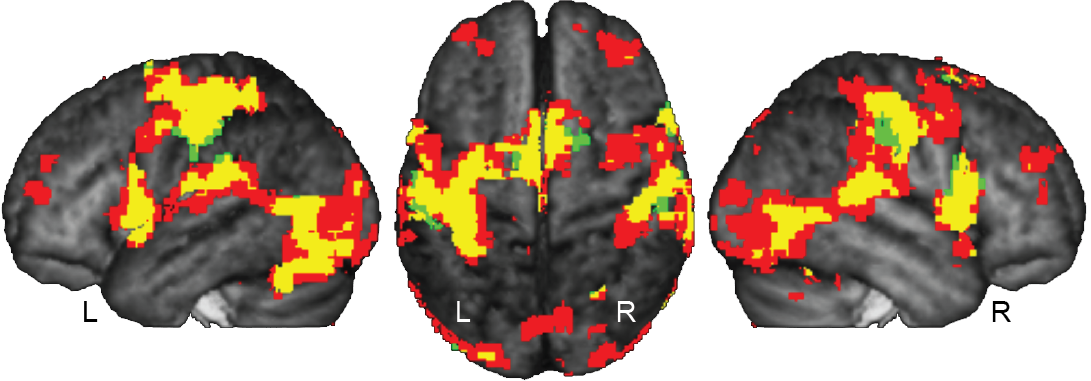


**Figure S5**: **PPIs group results.** *Green color:* second level PPI results currently presented in the manuscript (T<4.02 at p*_unc_*<0.001, all survive q*_fdr_* <0.05). The eigen-vectors were extracted from a 6mm sphere centered on the local maxima within the anatomical BA2 ROI. Eigen-vectors were extracted at the single subject level at p*_unc_* <0.001 for 14 out of 17 subjects. *Red color:* second level PPI results for the entire group of 17 subjects (T>3.69, p*_unc_* <0.001, all voxels also survive q*_fdr_*<0.05). As for green, the eigen-vectors were extracted from a 6mm sphere centered on the local maxima within the anatomical BA2 ROI. Eigen-vectors were extracted at single subject level within that region from all voxels where a subject showed a correlation with EMG, at p*_unc_*<0.001 threshold for 14 out of 17 subjects, and at p*_unc_*<0.5 for the remaining 4. *Yellow color:* overlap between Red and Green.

**
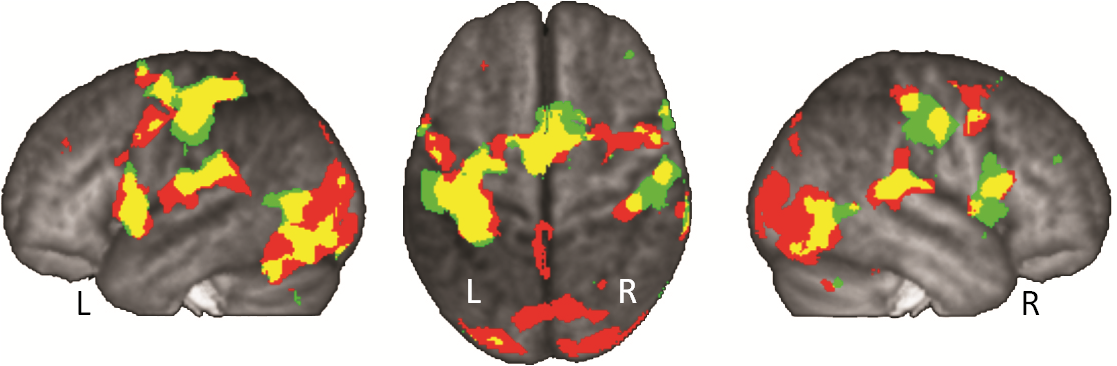
**

**Figure S6**: **PPIs group results.** *Green color:* second level PPI results currently presented in the manuscript (T<4.02 at p*_unc_*<0.001, all survive q*_fdr_* <0.05). The eigen-vectors were extracted from a 6mm sphere centered on the local maxima within the anatomical BA2 ROI. Eigen-vectors were extracted at the single subject level at p*_unc_*<0.001 for 14 out of 17 subjects. *Red color:* second level PPI results for the entire group of 17 subjects (T>3.69, p*_unc_*<0.001, all voxels also survive q*_fdr_*<0.05) using the method define above (supplementary method S4). *Yellow color:* overlap between Red and Green.

### **Supplementary Methods**

### Supplementary Method S1: Dynamic causal modeling (DCM)

Directed methods such as the DCM[1] attempt to derive the direction of information flow across regions. A limitation of DCM is the astronomic number of possible models to account for the connectivity pathways in even a limited number of regions of interest. Indeed, even with just five regions of interest, the number of possible connections between them and the possible task effects are in the order of many millions[2], orders of magnitude more than the number of data points acquired in typical fMRI experiments, making a systematic comparison impossible. Picking out a small number of models based on a priori hypotheses on the other hand becomes so arbitrary and subjective that it no longer satisfies the criteria for objectivity and reproducibility so important to science. In addition, simulations have shown that methods like DCM or Granger Causality, that aim to detect the direction of information flow in the brain, often detect directions of information that are opposite to the ones actually in the data[3,4]. We nevertheless tried to run two DCM analyses, with different numbers of regions of interests that were suggested by the PPI results, to disentangle whether differences between conditions depend on efference-copies from motor regions or re-afference from somatosensory cortices.

Based on the idea proposed by Haggard and Whitford[5] that the most likely source of the efference copy to somatosensory areas is the SMA, the first set of models only included the SMA, BA3b and BA2 regions of interest. The anatomical areas were again chosen from the cytoarchitectonic information provided by the Anatomy Toolbox 1.7 for SPM8, and again, due to the unilaterality of our task, which was only performed with the right hand, for BA2 and BA3b we only considered the left hemisphere. Subdividing SMA in right and left hemispheres is less justifiable since SMA covers the medial wall. Hence, as for the PPI analysis presented in the main text, SMA, was defined by including only the medial region (-17≤x≤17) of BA6. The eigen-vectors needed to run the DCM analysis were calculated at the first level by looking at the EMGpm>0 results with a threshold of p_unc_<0.05. This threshold was chosen to guarantee enough subjects in the analysis. Fourteen out of the seventeen subjects had significant voxels within the selected ROIs and they were therefore included in the final analysis. After defining candidate models, we used random effect Bayesian model selection implemented in SPM8 to identify the model with the highest evidence in the applied Bayesian framework. The investigated models and the results of this DCM analysis are shown in Figure S3. Results indicate that the efference-copy only model, in which the effect of EMG is introduced by the SMA is the most likely model. Models including an effect of EMG into BA3b, as would be the case if the somatosensory differences between high- and low-EMG trials were reaching BA2 through BA3b, are less likely.

In the second DCM analysis we included other possible sources of the efference-copy signal, in particular the premotor and the primary motor cortices. As Figure S4 illustrates, adding additional nodes tips the balance towards mixed models (similar to the results of our inverse covariance matrix analysis in the main paper). The winning model (model 9) is indeed one in which BA2 is influenced by a task dependent modulation from both BA3b and the motor regions. The next-best model however, is of much lower complexity (making the comparison difficult), and includes a direct effect of EMG on BA2 that is difficult to interpret. This result raises many questions, about the role complexity plays in the Bayesian comparison, and about whether variants of the models would win[2], making DCM ill suited for our purpose. We therefore do not explore DCM further for this data-set.

For the main paper, we therefore felt that an approach that can accommodate all relevant ROIs, and that can explore the whole search space of possible connections and task effects in a principled and exhaustive manner would be preferable to an arguably arbitrary a priori selection of models. Because Lohmann et al[2], essentially point out that DCM cannot do that, and because Smith et al.[4] came to the conclusion that methods based on partial correlation are the most robust methods to explore connectivity from fMRI data, we opted to use partial correlations as our main tool in the main manuscript. Reasuringly, partial correlations confirmed the tentative conclusion of our DCM analyses, namely that an input to BA2 from motor structures is significant, and cannot be reduced to re-afference from BA3b alone.

# Supplementary method S2: Influence of the EEG artifact on the partial correlation analyses

**A**s mentioned in the method section, during the whole experimental session, subjects wore an EEG cap. This is because the data presented here were collected together with the data presented in Arnstein et al. 2011[6], for which the EEG was required. Unexpectedly, the bundle of cables connecting the EEG cap to the amplifier, which was often pressed close to the subject’s scalp by the MRI head coil, was the likely source of the drop in BOLD signal intensity over the left parietal lobe observed in most subjects. These reduced EPI signals lead SPM8 to consider these voxels out of the brain. To solve this problem: (a) all subjects’ smoothed and normalized mean EPIs were averaged into a grand mean EPI; (b) this grand mean EPI was divided by each subject’s mean EPI; (c) we then multiplied, for each subject separately, all the smoothed EPIs by the subject's correction map obtained in point (b) (see Supplementary Fig. 1 from Arnstein et al. (2011) for more details, http://www.herseninstituut.knaw.nl/Portals/0/Department/keysers/Arnstein%20SupplementaryFigures.pdf). This procedure allowed SPM8 to accurately identify the boundaries of the left parietal lobe for all subjects. While this procedure does not bias the significance of the univariate approach of the General Linear Model that examines changes over time, because the same correction factor was applied to all EPI images of the same participant, it might affect the connectivity analyses run on the data presented in the current manuscript. Because we explore efference-copy signals using partial correlations between BA2 and five motor control ROIs after removing the effect of task and BA3b, if dropout would bias these connectivity measures, we would expect a correlation across participants: the amount of dropout in a participant should predict the value of the connectivity measure between the ROIs in that participant. We thus explored whether individual differences in signal drop-out in BA2 predict individual differences in the partial correlation between BA2 and any of the motor control ROIs (SMA, PM, M1, Cerebellum and PF). We found that in none of these cases, the correlation between the dropout in BA2 (quantified as the average correction index within the BA2 ROI) and the connectivity measure (partial correlation BA2 and motor control ROI after taking out the effect of task and BA3b) was significant. The five correlations ranged from r=.33 to r=.18, with p values ranging from p=0.25 to p=0.51. Also, no significant correlations were found, when using the dropout in a motor control ROI to predict the partial correlation between that region and BA2. This suggests that our connectivity analyses were not systematically biased by dropout over BA2 or any of the motor control ROIs. There was also no linear relationship between the variance (over time) of the ROI time course in BA2 (after correction) and the average drop-out in that ROI (p>0.83).

# Supplementary method S3: PPI analysis including all 17 subjects

To be able to include the data (i.e. eigen-vector) from all our participants, and therefore have results that, although most likely influenced by noise, are more representative of the group, we re-run the PPI analysis by lowering the single subjects threshold of the EMGpm>0 single subject results used to identify the local maxima within our left BA2 region of interest. For the 4 subjects at which there were no voxels left at p*_unc_*<0.001 we therefore used p*_unc_*<0.5. The results of the second level of the analysis, run in the same way of the analysis presented in the main text, are shown in Figure S5 and are very similar to the main results (both at p<0.001 also surviving p*_fdr_*<0.05).

**Supplementary Method S4: PPI results using group-level results to define ROIs.**

The BA2 ROI was defined as follows. First, left BA2 was defined using the cytoarchitectonic information provided by the Anatomy Toolbox 1.7[7,8] for SPM8. Next, the portion of this anatomical region that correlated with the EMG (p*_unc_*<0.001, k>10) at the second-level was used to make a binary mask. Finally, the first eigenvariate was extracted for the voxels in this mask that were activated in the first-level EMG>0 results (p*_unc_*<0.05 uncorrected, k>0, or, if fewer than 50 voxels were activated at p*_unc_*<0.05, p*_unc_*<0.5 was used) for each subject. Note that these loose statistical thresholds were used only to define ROIs, not to make population inferences.

Again, the results of the second level of the analysis (Figure S6, p*_unc_*<0.001 also surviving p*_fdr_*<0.05), were run in the same way and resulted very similar to the analysis presented in the main text.

Reference List

1. Friston KJ, Harrison L, Penny W (2003) Dynamic causal modelling. Neuroimage 19: 1273-1302. S1053811903002027 [pii].

2. Lohmann G, Erfurth K, Muller K, Turner R (2012) Critical comments on dynamic causal modelling. Neuroimage 59: 2322-2329. S1053-8119(11)01071-8 [pii];10.1016/j.neuroimage.2011.09.025 [doi].

3. Schippers MB, Renken R, Keysers C (2011) The effect of intra- and inter-subject variability of hemodynamic responses on group level Granger causality analyses. Neuroimage 57: 22-36. S1053-8119(11)00146-7 [pii];10.1016/j.neuroimage.2011.02.008 [doi].

4. Smith SM, Miller KL, Salimi-Khorshidi G, Webster M, Beckmann CF, Nichols TE, Ramsey JD, Woolrich MW (2011) Network modelling methods for FMRI. Neuroimage 54: 875-891. S1053-8119(10)01160-2 [pii];10.1016/j.neuroimage.2010.08.063 [doi].

5. Haggard P, Whitford B (2004) Supplementary motor area provides an efferent signal for sensory suppression. Brain Res Cogn Brain Res 19: 52-58. 10.1016/j.cogbrainres.2003.10.018 [doi];S0926641003002854 [pii].

6. Arnstein D, Cui F, Keysers C, Maurits NM, Gazzola V (2011) mu-suppression during action observation and execution correlates with BOLD in dorsal premotor, inferior parietal, and SI cortices. J Neurosci 31: 14243-14249. 31/40/14243 [pii];10.1523/JNEUROSCI.0963-11.2011 [doi].

7. Eickhoff SB, Stephan KE, Mohlberg H, Grefkes C, Fink GR, Amunts K, Zilles K (2005) A new SPM toolbox for combining probabilistic cytoarchitectonic maps and functional imaging data. Neuroimage 25: 1325-1335. S1053-8119(04)00792-X [pii];10.1016/j.neuroimage.2004.12.034 [doi].

8. Grefkes C, Geyer S, Schormann T, Roland P, Zilles K (2001) Human somatosensory area 2: observer-independent cytoarchitectonic mapping, interindividual variability, and population map. Neuroimage 14: 617-631. 10.1006/nimg.2001.0858 [doi];S1053-8119(01)90858-4 [pii].
